# Optimized Irrigation and Fertilization Reduce Luxury Transpiration While Improving GRAIN Yield, Water Use Efficiency, and Economic Benefits of Winter Wheat in the Arid Region of Xinjiang

**DOI:** 10.3390/plants15111629

**Published:** 2026-05-26

**Authors:** Zhiying Liu, Liang Cheng, Yannian Li, Liaoyuan Ma, Wangyang Li, Tao Sun, Jinqi Wu, Shiqi Liu, Ruiqi Du, Zijun Tang, Fucang Zhang, Youzhen Xiang

**Affiliations:** 1 College of Water Resources and Architectural Engineering, Northwest A&F University, Yangling 712100, China; 2024055914@nwafu.edu.cn (Z.L.); chengliang2@nwafu.edu.cn (L.C.); liyannian7@nwafu.edu.cn (Y.L.); 2023012608@nwafu.edu.cn (L.M.); lwy1222@nwafu.edu.cn (W.L.); 2021050986@nwafu.edu.cn (T.S.); 2025056093@nwafu.edu.cn (J.W.); 2025056042@nwafu.edu.cn (S.L.); tangzijun@nwsuaf.edu.cn (Z.T.); 2 Xinjiang Research Institute of Agriculture in Arid Areas, Urumqi 830091, China; 3 Key Laboratory of Agricultural Soil and Water Engineering in Arid and Semiarid Areas of Ministry of Education, Northwest A&F University, Yangling 712100, China; 4 College of Geomatics, Xi’an University of Science and Technology, Xi’an 710054, China; duruiqi@126.com; 5 Key Laboratory of Northwest Oasis Water-Saving Agriculture, Ministry of Agriculture and Rural Affairs, Xinjiang Academy of Agricultural and Reclamation Science, Shihezi 832000, China; zhangfc@nwsuaf.edu.cn

**Keywords:** winter wheat, water-fertilizer coupling, luxury transpiration, water use efficiency, multi-objective optimization

## Abstract

Winter wheat production in the extremely arid oasis region of Xinjiang relies heavily on irrigation and fertilization, but conventional high-input management can induce luxury transpiration and non-productive water consumption, limiting the coordinated improvement of grain yield, water use efficiency (WUE), and economic benefits. To identify the threshold at which water–fertilizer inputs shift from efficient use to inefficient water consumption and to define a robust management range, a two-year field experiment was conducted in southern Xinjiang during the 2022–2023 and 2023–2024 growing seasons. Four irrigation levels, corresponding to 60%, 80%, 100%, and 120% of crop evapotranspiration (*ET_c_*), and four fertilization levels were established to evaluate the effects of water–fertilizer interactions on canopy development, leaf gas exchange, evapotranspiration, yield, WUE, and economic benefits. Appropriate water and nutrient supply promoted canopy establishment and maintained higher photosynthetic capacity, thereby increasing grain yield, WUE, and net return. However, excessive inputs weakened yield gains and failed to synchronously improve WUE and economic benefits. Linear plateau models revealed clear thresholds in both the crop-stand scale evapotranspiration (*ET*)–dry matter accumulation (DM) relationship and the leaf-scale transpiration rate (*Tr*)–net photosynthetic rate (*Pn*) relationship. The seasonal *ET* thresholds were 504.59 and 553.87 mm in the two growing seasons, respectively, and the *Tr* threshold was 4.83 mmol m^−2^ s^−1^. Beyond these thresholds, additional water consumption was not effectively converted into photosynthetic assimilation or biomass accumulation, indicating luxury transpiration. Year-specific response surface analysis and TOPSIS evaluation showed that I3F3, namely 100% *ET_c_* combined with 210–195–75 kg ha^−1^ N–P_2_O_5_–K_2_O, together with its adjacent range, sustained high grain yield, WUE, and economic benefits, with I3F3 achieving the best overall performance in both years. The intersection of the two-year high-performance regions further defined a robust interannual feasible range with an irrigation amount of 506.21–545.09 mm and a total fertilizer input of 369.54–628.33 kg ha^−1^. Overall, maintaining water and fertilizer inputs within the I3F3-adjacent range can reduce non-productive water consumption and luxury transpiration risk while synergistically improving grain yield, WUE, and economic benefits in winter wheat.

## 1. Introduction

Wheat (*Triticum aestivum* L.) is one of the major staple crops worldwide, serving as the primary food source for approximately 35–40% of the global population and playing a crucial role in ensuring global food security and maintaining the stability of agricultural trade [1]. Xinjiang is an important strategic grain reserve base and a major wheat-producing region in China; however, agricultural production in this region has long been constrained by the uneven spatiotemporal distribution of water resources [2]. This constraint is particularly pronounced in the extremely arid oasis regions of southern Xinjiang, where the mean annual precipitation is only approximately 60 mm, whereas potential evaporation exceeds 2000 mm [3]. As a result, winter wheat production is highly dependent on irrigation and fertilization inputs. Driven by strong evaporative demand and the need to maintain stable and high yields, high-irrigation and high-fertilization management practices are widely adopted in local production systems. Although this approach can alleviate water and nutrient limitations to some extent, it is often accompanied by increased water consumption, reduced fertilizer use efficiency, and higher production costs [4]. Therefore, developing precise and efficient water–fertilizer management strategies for winter wheat production in arid regions is of great importance for ensuring regional food security and promoting sustainable agricultural development.

Irrigation and fertilization are key agronomic practices regulating winter wheat canopy establishment, photosynthetic production, and yield formation. Adequate water supply maintains leaf turgor and stomatal opening, thereby promoting leaf area expansion, canopy development, and dry matter accumulation. In contrast, water deficit induces stomatal closure, suppresses carbon assimilation and grain filling, and ultimately constrains yield formation [5,6]. Appropriate fertilization facilitates chlorophyll synthesis, sustains leaf photosynthetic activity, and supports the coordinated transition from vegetative to reproductive growth, providing a fundamental basis for achieving high yield and high resource-use efficiency [7,8]. However, water and nutrients do not act independently; instead, they interact through complex coupling mechanisms [9,10]. Nutrient uptake and transport are highly dependent on water availability, whereas the beneficial effects of irrigation can be fully realized only when supported by sufficient and balanced nutrient supply [11]. Previous studies have shown that winter wheat responses to water and fertilizer inputs are generally characterized by pronounced nonlinearity and threshold effects, indicating that excessive inputs do not continuously generate proportional improvements in crop growth or grain yield [12].

The diminishing gains observed under high-input management may be closely associated with the occurrence of “luxury transpiration” within the crop system. Luxury transpiration does not simply refer to a high transpiration rate, but rather to an inefficient water consumption process in which additional water use can no longer be effectively converted into photosynthetic assimilation, dry matter accumulation (DM), or yield benefits. At the leaf scale, appropriate water supply combined with adequate nutrient availability generally helps maintain favorable leaf photosynthetic performance and gas exchange. However, when water and fertilizer inputs exceed the level required for efficient crop utilization, increases in transpiration rate (*Tr*) are not necessarily accompanied by synchronous improvements in net photosynthetic rate; under strong evaporative demand, *Tr* may remain high or even continue to increase with enhanced water supply, whereas the increase in net photosynthetic rate (*Pn*) gradually diminishes and tends to plateau. Consequently, additional water consumption beyond the critical range does not necessarily generate proportional gains in photosynthetic carbon assimilation [13,14]. At the crop-stand scale, appropriate water and nutrient supply favors canopy development and improves light interception, but excessive inputs may result in excessive leaf area expansion, intensified within-canopy shading, increased maintenance costs of the canopy, and a greater proportion of soil evaporation, causing additional water consumption to shift gradually from efficient productive use to inefficient loss [15,16]. Thus, luxury transpiration essentially reflects the decoupling between increased water consumption and assimilate accumulation under high water and fertilizer inputs. Once water and nutrient supply exceeds the threshold for efficient crop utilization, additional water use is difficult to translate effectively into photosynthetic assimilation and dry matter accumulation, thereby weakening its contribution to the coordinated improvement of grain yield, WUE, and economic benefits. However, most studies on water–fertilizer regulation have mainly focused on comparing the responses of leaf photosynthesis, crop evapotranspiration (*ET*), and yield among different irrigation and fertilization treatments. Few studies have integrated the leaf-scale *Tr*–*Pn* relationship with the crop-stand-scale *ET*–DM relationship to systematically identify the critical thresholds for the onset of luxury transpiration. Consequently, it remains difficult to determine when additional water and fertilizer inputs shift from efficient utilization to inefficient water consumption [17,18].

The optimization of water and fertilizer management in winter wheat is challenging because grain yield, WUE, and economic benefits all exhibit pronounced nonlinear responses to water and nutrient inputs, and these three objectives do not always improve synchronously in practical agricultural production. A sole pursuit of high yield may increase water consumption and input costs, thereby compromising water-saving performance and economic profitability [19]. In arid agricultural systems, interannual meteorological variability can substantially alter crop evapotranspiration demand, resource use efficiency, and production returns, thereby affecting the applicability and stability of water–fertilizer management strategies [20,21]. Therefore, optimizing water and fertilizer management for winter wheat in arid regions requires not only balancing the trade-offs among yield, WUE, and economic benefits, but also identifying water–fertilizer management ranges that remain highly stable across different growing seasons. Response surface methodology (RSM) can quantitatively characterize the continuous nonlinear relationships among irrigation, fertilization, and crop response variables, making it suitable for identifying high-performance water–fertilizer feasible domains across growing seasons [22]. Further extracting the intersection of the high-performance regions from two consecutive years can provide a water–fertilizer management range with greater interannual stability and field applicability. On this basis, TOPSIS-based comprehensive evaluation enables multi-criteria ranking of measured water–fertilizer treatments and facilitates the selection of representative optimal treatments in terms of yield, WUE, and economic benefits. Integrating the identification of luxury transpiration thresholds, response surface-based range analysis, and comprehensive evaluation of measured treatments can provide a more systematic basis for defining the efficient water–fertilizer utilization range for winter wheat production in arid regions.

Based on these considerations, this study was conducted using a two-year field experiment with irrigation and fertilization gradients in a typical extremely arid oasis region of southern Xinjiang to systematically investigate the interactive effects of water and fertilizer inputs on canopy establishment, leaf gas exchange, evapotranspiration, and production benefits of winter wheat. We hypothesized that a critical range exists in winter wheat production, beyond which water and fertilizer inputs shift from efficient utilization to inefficient water consumption. This transition can be characterized by plateau thresholds in the leaf-scale *Tr*–*Pn* relationship and the crop-stand-scale *ET*–DM relationship, and water–fertilizer combinations near these thresholds are more likely to reduce the risk of luxury transpiration while simultaneously improving grain yield, WUE, and economic benefits. The specific objectives were to: (1) clarify the regulatory effects of irrigation and fertilization on canopy establishment, evapotranspiration, leaf chlorophyll content, and gas exchange parameters of winter wheat; (2) reveal the coupling relationship and threshold characteristics of luxury transpiration at the crop-stand and leaf scales; and (3) determine a robust water–fertilizer management range that consistently achieves high yield, high WUE, and high economic benefits across two consecutive growing seasons using response surface methodology, and select representative optimal treatments through TOPSIS-based comprehensive evaluation. The results are expected to provide a theoretical basis and practical reference for shifting winter wheat production in the arid regions of southern Xinjiang from a high-input yield-increasing strategy toward precise and efficient water–fertilizer regulation.

## 2. Materials and Methods

### 2.1. Study Site

**Figure 1:**
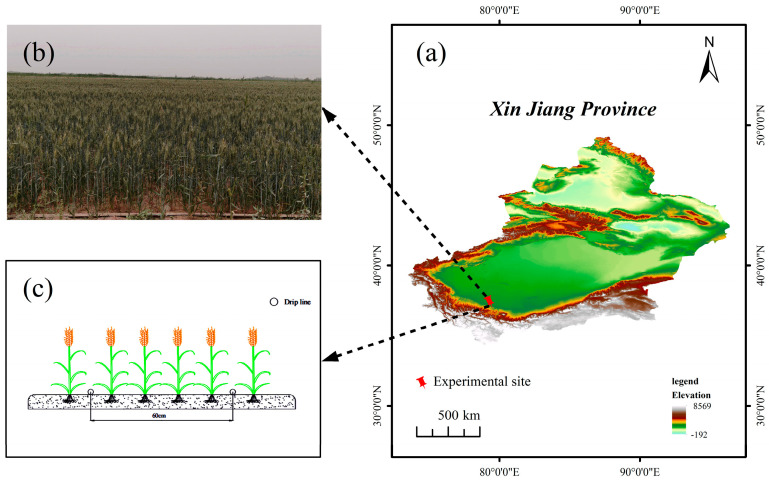
Overview of the experimental site and drip-irrigated winter wheat planting pattern: (**a**) Location and elevation of the experimental site; (**b**) field view of winter wheat; (**c**) schematic diagram of drip-irrigated planting pattern.

**Figure 2:**
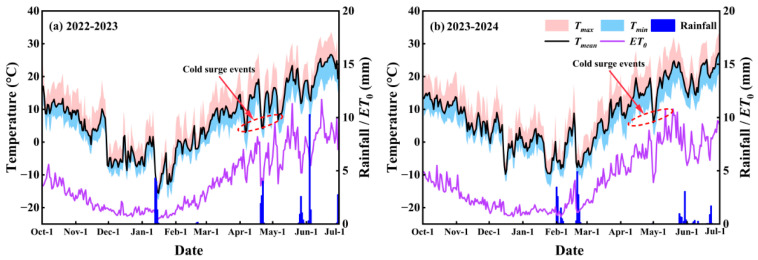
Meteorological conditions during the winter wheat growing seasons of (**a**) 2022–2023 and (**b**) 2023–2024. T_max_, T_min_, and T_mean_ represent the daily maximum, minimum, and mean air temperatures, respectively. *ET*_0_ represents the daily reference crop evapotranspiration. The red dashed ellipses highlight the occurrence of spring cold wave events.

The field experiment was conducted during the 2022–2023 and 2023–2024 winter wheat growing seasons in Kunyu City, the 14th Division of the Xinjiang Production and Construction Corps, China (37°36′ N, 79°35′ E; 1365 m above sea level) (Figure 1). The study area has a warm temperate continental arid climate and is characterized by extreme aridity, with annual precipitation of only 20–60 mm and annual evaporation as high as 2240 mm. The mean annual air temperature is 14.7 °C, the annual sunshine duration is 2650–2800 h, and the frost-free period is approximately 240 days. During the 2022–2023 and 2023–2024 winter wheat growing seasons (from October of the previous year to June of the following year), the maximum temperatures were 36.25 °C and 36.52 °C, respectively, while the minimum temperatures were −17.73 °C and −13.92 °C, respectively. Total effective precipitation from sowing to harvest was 30.3 mm in 2022–2023 and 23.9 mm in 2023–2024 (Figure 2). Reference evapotranspiration (*ET*_0_), estimated using the FAO-56 Penman–Monteith equation based on daily meteorological data, was 8.9% lower in the 2022–2023 growing season than in 2023–2024, mainly due to two additional cold-wave events, defined as temperature drops of ≥10 °C within 48 h. The soil at the experimental site was sandy loam, with an average bulk density of 1.66 g cm^−3^ and a field capacity of 18.87% in the 0–140 cm soil layer. The physicochemical properties of the soil at 0–30 cm depth are presented in Table 1.

### 2.2. Experimental Design

A two-factor randomized block design was adopted in this study, with four irrigation levels and four fertilization gradients. The irrigation treatments were designed according to the calculated crop evapotranspiration (*ET_c_*), which was obtained from reference evapotranspiration (*ET*_0_) estimated using the FAO-56 Penman–Monteith equation and the crop coefficient of winter wheat. Accordingly, I3, corresponding to 100% *ET_c_*, was used as the full-irrigation reference level representing the calculated crop water requirement, while I1, I2, and I4 represented 60%, 80%, and 120% *ET_c_*, respectively. The fertilization gradients were established based on the regional N:P_2_O_5_:K_2_O ratio of 14:13:5. Among them, F3, corresponding to 210–195–75 kg ha^−1^ N–P_2_O_5_–K_2_O, was selected to represent the conventional local fertilization rate based on a preliminary survey of winter wheat production practices in the study area. The other fertilizer levels, namely F1: 70–65–25, F2: 140–130–50, and F4: 280–260–100 kg ha^−1^ N–P_2_O_5_–K_2_O, were set as lower or higher input gradients relative to F3. Urea (46% N), diammonium phosphate (18% N and 46% P_2_O_5_), and potassium sulfate (52% K_2_O) were used as fertilizer sources. A total of 16 treatment combinations were established, each with three replicates, resulting in 48 experimental plots in total. Each plot measured 20 m × 10 m, and adjacent plots were separated by 0.8 m-wide physical barriers to prevent lateral water movement between plots.

Winter wheat cultivar Xindong 44 was sown on 17 October 2022 and 20 October 2023, respectively. The crop entered the green-up stage on 1 March 2023 and 5 March 2024, and was finally harvested on 15 June 2023 and 20 June 2024, respectively. No significant incidence of diseases or insect pests requiring plant protection intervention was observed during either growing season.

Calculated crop evapotranspiration in this study was estimated using the following equation [23]:

(1) $${ET}_{c}=k_{c}\times {ET}_{0}$$

(2) $${ET}_{0}=\frac{0.408\Delta \left(R_{n}-G\right)+\gamma \frac{900u_{2}\left(e_{s}-e_{a}\right)}{T_{mean}+273}}{\Delta +\gamma \left(1+0.34u_{2}\right)}$$

where *ET_c_* is calculated crop evapotranspiration (mm d^−1^); *k_c_* is the crop coefficient of winter wheat, with values of 0.4 during the sowing-seedling stage, 1.1 during the jointing-milking stage, and 0.4 during the wax ripening-harvest stage; *ET*_0_ is reference crop evapotranspiration (mm d^−1^); *R_n_* is net radiation at the crop surface (MJ m^−2^ d^−1^); *e_s_* − *e_a_* is the saturation vapour pressure deficit (kPa); Δ is the slope of the saturation vapour pressure curve; γ is the psychrometric constant (kPa °C^−1^); *G* is soil heat flux (MJ m^−2^ d^−1^); and *u*_2_ is wind speed at 2 m height (m s^−1^).

**Figure 3:**
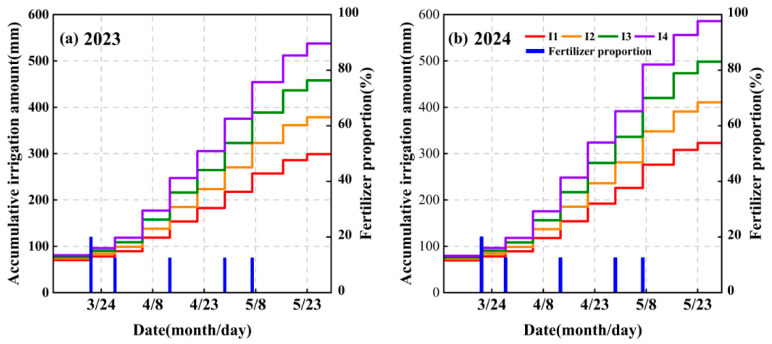
Schematic diagram of the timing and amount dynamics of drip irrigation and fertilization for winter wheat in (**a**) 2023 and (**b**) 2024. The stepwise lines represent the cumulative irrigation amounts under different irrigation levels (I1–I4), and the blue bars indicate the fertilizer proportions applied at each topdressing stage.

In this experiment, an integrated water-fertilizer management strategy combining a differential-pressure fertilizer tank with a drip irrigation system was adopted. The system was equipped with drip tapes of 1.6 cm outer diameter, operated at a working pressure of 0.1 MPa, with emitters spaced at 30 cm and a discharge rate of 2 L h^−1^. The drip tapes were uniformly arranged along the centerline of the wheat rows, with an equal spacing of 60 cm between adjacent drip lines (Figure 3). Seasonal irrigation started at the green-up stage and ended on May 23 of each year. Irrigation was applied at approximately 7-day intervals, and the amount for each irrigation event was determined according to the 7-day cumulative crop evapotranspiration. Including the initial irrigation at the green-up stage, irrigation was applied 10 times during each growing season. In 2022–2023, the seasonal irrigation amounts for treatments I1, I2, I3, and I4 were 298.8, 378.4, 458.0, and 537.5 mm, respectively. In 2023–2024, the corresponding irrigation amounts increased by 5.3–9.3%, reaching 323.0, 410.7, 498.3, and 586.0 mm for I1, I2, I3, and I4, respectively. Fertilizer was applied using a split-application strategy: 30% was incorporated as basal fertilizer before sowing, and the remaining 70% was fertigated in five equal or staged applications through the drip irrigation system, including 20% at green-up, 12.5% at jointing, 12.5% at booting, 12.5% at anthesis, and 12.5% at grain filling (Figure 3). Urea (46% N), diammonium phosphate (18% N and 46% P_2_O_5_), and potassium sulfate (52% K_2_O) were used as fertilizer sources.

### 2.3. Measurement Methods

#### 2.3.1. Dry Matter Accumulation

At the key phenological stages of wheat, including green-up, jointing, anthesis, grain filling, and maturity, representative wheat plants were sampled from a 1 m row length in each plot and cut neatly at the stem base. The plant samples were first heated at 105 °C for 30 min to deactivate enzymes, and then oven-dried at a constant temperature of 75 °C until a constant weight was reached.

A logistic equation was used to characterize the response of dry matter accumulation (DM) under different treatments, and the equation was expressed as follows [24]:

(3) $$y=\frac{k}{1+ae^{-bt}}$$

where *y* is the dry matter accumulation of winter wheat (kg ha^−1^); *k* is the theoretical maximum dry matter accumulation (kg ha^−1^); *t* is the number of days after sowing; and a and b are model parameters.

The characteristic parameters were calculated as follows:

(4) $$t_{1}=\frac{1}{b}ln\left(\frac{a}{2+\sqrt{3}}\right)$$

(5) $$t_{2}=\frac{1}{b}ln\left(\frac{a}{2-\sqrt{3}}\right)$$

(6) $$t_{d}=t_{2}-t_{1}$$

where *t*_1_ and *t*_2_ are the starting and ending times of the rapid growth period, respectively (d), and *t_d_* is the duration of the rapid growth period (d).

#### 2.3.2. Leaf Area Index

Leaf area index (LAI) was estimated using a modified punch method combined with specific leaf area (SLA). LAI was measured five times during the growing season, namely at the green-up, jointing, anthesis, grain-filling, and maturity stages. At each measurement stage, representative plants were sampled from each plot. For each plant, 10 fresh leaves were randomly selected from different positions of the plant canopy, and circular discs were uniformly sampled with a 5 mm-diameter punch while avoiding the leaf midrib; 10 discs were taken from each leaf. The total number of discs was recorded, and the total disc area (*A_disc_*, m^2^) was calculated. The sampled discs were then oven-dried at 75 °C for 48 h to constant weight, after which their dry weight (*W_disc_*, kg) was measured. Specific leaf area (SLA, m^2^ kg^−1^), defined as the ratio of leaf area to leaf dry mass, was calculated as follows [25,26]:

(7) $$SLA=\frac{A_{disc}}{W_{disc}}$$

The total leaf dry mass per plant (*W_plant_*, kg) was determined by the oven-drying method. Based on the proportional relationship between measured leaf area and dry weight described above, the leaf area per plant (*A_plant_*, m^2^) was calculated using the following equation:

(8) $$A_{plant}=SLA\times W_{plant}$$

LAI was defined as the one-sided leaf area per unit ground area and was calculated as follows [27]:

(9) $$LAI=\frac{A_{plant}}{A_{ground}}$$

where *A_ground_* is the ground area occupied by each plant. This method provides an indirect estimate of plant leaf area based on representative leaf subsamples and total leaf dry mass. Although variation in SLA among leaf positions may introduce some uncertainty, the same sampling and calculation procedure was applied consistently across all treatments and growth stages, allowing reliable comparison of relative treatment differences.

#### 2.3.3. Grain Yield

At the maturity stage of winter wheat, three 1 m^2^ (1 m × 1 m) quadrats were selected from each experimental plot, and grain yield was determined by manual threshing. The measured yield was then standardized to a grain moisture content of 14%.

#### 2.3.4. Leaf Chlorophyll Content and Gas Exchange Characteristics

Leaf chlorophyll content (*C_ab_*) was estimated from non-destructive SPAD measurements using a portable chlorophyll meter SPAD-502 (Konica Minolta, Tokyo, Japan). SPAD measurements were conducted four times during the growing season, namely at the green-up, jointing, anthesis, and grain-filling stages. At each measurement stage, 10 uniformly growing plants were randomly selected from each plot. For each plant, the three uppermost fully expanded leaves were measured using the SPAD meter, and the readings were averaged to obtain the plot mean. Although SPAD readings are dimensionless, they provide a rapid indication of relative leaf chlorophyll status and leaf greenness [28]. Subsequently, the SPAD readings were converted to Cab values following the calibration method established by Markwell et al. [29].

(10) $$\mathrm{Chlorophyll}\;(\mu g\cdot {\mathrm{cm}}^{-2})=0.0893\times {\;10}^{{SPAD}^{0.256}}$$

Leaf net photosynthetic rate (*Pn*, μmol·m^−2^·s^−1^) and transpiration rate (*Tr*, mmol·m^−2^·s^−1^) were measured using a portable photosynthesis system LI-6400XT (LI-COR Biosciences, Lincoln, NE, USA). Measurements were conducted under standardized environmental conditions, with photosynthetic photon flux density (PPFD) set to 1500 μmol·m^−2^·s^−1^, CO_2_ concentration at 400 μmol·mol^−1^, and leaf temperature maintained at 28 ± 0.5 °C. All measurements were performed on clear, windless days between 10:30 and 13:30 local time. The gas exchange measurements were conducted on the same dates and at the same growth stages as the SPAD measurements, and the selected leaves corresponded to those used for SPAD-based chlorophyll assessments.

Instantaneous photosynthetic water use efficiency (PWUE) was calculated as the ratio of *Pn* to *Tr* [30]:

(11) $$PWUE=\frac{P_{n}}{T_{r}}$$

#### 2.3.5. Water Use Efficiency

Water use efficiency (WUE) is an index describing the relationship between crop yield and water consumption, and reflects the ability of the crop to convert water into yield. WUE was calculated as follows:

(12) $$WUE=\frac{GY}{ET\times 10}$$

where *GY* is the grain yield of winter wheat (kg ha^−1^), and *ET* is the crop water consumption (mm).

Actual crop evapotranspiration (*ET*) was calculated as follows [31]:

(13) ET=P+I+U−D−R−ΔS

where *P* is effective precipitation (mm); *U* is groundwater recharge (mm); *I* is irrigation amount (mm); *R* is surface runoff (mm); *D* is deep drainage (mm); and Δ*S* is the change in soil water storage from the beginning to the end of the experiment (mm). Under the experimental conditions, *U*, *D*, and R were assumed to be negligible.

#### 2.3.6. Economic Benefit

The economic benefit of winter wheat was evaluated using net economic benefit, defined as the difference between gross income and total cost. Gross income was calculated based on grain yield and the grain price in the corresponding year, whereas total cost included seed cost, water charges, fertilizer cost, and other expenses, such as drip irrigation equipment investment and labor cost. Net economic benefit was calculated as follows:

(14) $$G_{p}=GY\times P_{g}$$

(15) $$E_{b}=G_{p}-I_{s}-I_{w}-I_{f}-I_{O}$$

where $G_{p}$ is the gross grain income (CNY ha^−1^); $P_{g}$ is the grain price in the corresponding year (CNY kg^−1^) *E_b_* is the net economic benefit (CNY ha^−1^); *I_s_* is the seed input cost (CNY ha^−1^); *I_w_* is the irrigation water cost (CNY ha^−1^); *I_f_* is the fertilizer input cost (CNY ha^−1^); and *I_o_* is the other input costs (CNY ha^−1^).

#### 2.3.7. Linear Plateau Threshold Analysis

To identify the critical thresholds for the onset of luxury transpiration, namely the breakpoints beyond which additional water consumption was no longer effectively converted into dry matter accumulation or increases in net photosynthetic rate, linear plateau models were used to fit the *ET*–DM relationship at the crop stand scale and the *Tr*–*Pn* relationship at the leaf scale. The model was expressed as follows [32]:

(16) $$y=\left\{\begin{array}{cc} a+bx, & x\leq c\\ a+bc, & x>c \end{array}\right.$$

where *y* is the response variable, *x* is the explanatory variable, a is the intercept, b is the slope before the breakpoint, and c is the breakpoint of the model, which was defined as the threshold for luxury transpiration in this study. At the crop-stand scale, *x* represented seasonal evapotranspiration (*ET*, mm), and *y* represented dry matter accumulation at maturity (DM, Mg ha^−1^). The *ET*–DM relationship was fitted separately for the 2022–2023 and 2023–2024 growing seasons. At the leaf scale, *x* represented transpiration rate (*Tr*, mmol m^−2^ s^−1^), and *y* represented net photosynthetic rate (*Pn*, μmol m^−2^ s^−1^), using measurements from the anthesis and grain-filling stages. The parameters a, b, and c were estimated simultaneously using nonlinear least-squares regression, and the value of c that minimized the residual sum of squares was taken as the threshold. The corresponding plateau value was calculated as a+bc. Model performance was evaluated using the coefficient of determination (R^2^) and significance tests.

#### 2.3.8. Response Surface Methodology

To quantitatively describe the non-linear responses of winter wheat yield, water use efficiency (WUE), and economic benefit to irrigation and fertilization, and to provide continuous objective functions for subsequent robust optimization, quadratic polynomial response surface models were constructed based on two growing seasons of field trial data. Irrigation amount (*I*, mm) and fertilizer application rate (*F*, kg ha^−1^) were used as independent variables, while yield, WUE, and economic benefit served as the response variables [33]:

(17) $${\hat{Z}}_{i}(I,F)=\beta_{0,i}+\beta_{1,i}I+\beta_{2,i}F+\beta_{12,i}IF+\beta_{11,i}I^{2}+\beta_{22,i}F^{2}$$

where ${\hat{Z}}_{i}$ is the predicted response for the i-th growing season; where i = 1 and 2 correspond to the 2022–2023 and 2023–2024 growing seasons, respectively; Z denotes grain yield, WUE, or economic benefit. The coefficients $\beta_{0,i}$, $\beta_{1,i}$, $\beta_{2,i}$, $\beta_{12,i}$, $\beta_{11,i}$, and $\beta_{22,i}$ are regression parameters. Model performance was evaluated using the coefficient of determination (R^2^), adjusted R^2^ (Adj-R^2^), and significance tests.

#### 2.3.9. TOPSIS-Based Comprehensive Evaluation

To further identify representative optimal treatments that simultaneously achieved high yield, high WUE, and high economic benefits among the 16 measured water–fertilizer treatments, the TOPSIS method was used for comprehensive evaluation. The evaluation indicators included grain yield, WUE, and net economic benefit, all of which were regarded as benefit-type indicators. First, vector normalization was performed for each indicator, and a weighted normalized decision matrix was then constructed. Subsequently, the positive ideal solution and negative ideal solution were determined, and the distances of each treatment from the positive and negative ideal solutions, as well as the relative closeness coefficient, were calculated. The calculation procedure was as follows [34]:

(18) $$Z_{mv}=w_{v}\frac{x_{mv}}{\sqrt{\sum_{m=1}^{M}x_{mv}^{2}}}$$

(19) $$D_{m}^{+}=\sqrt{\sum_{v=1}^{V}{\left(Z_{mv}-Z_{v}^{best}\right)}^{2}}$$

(20) $$D_{m}^{-}=\sqrt{\sum_{v=1}^{V}{\left(Z_{mv}-Z_{v}^{worst}\right)}^{2}}$$

(21) $$C_{m}=\frac{D_{m}^{-}}{\left(D_{m}^{+}+D_{m}^{-}\right)}$$

where $x_{mv}$ denotes the observed value of the mth water–fertilizer treatment for the vth evaluation indicator; $Z_{mv}$ is the weighted normalized value; M is the number of treatments, with M=16 in this study; and V is the number of evaluation indicators, with V=3, namely grain yield, WUE, and net economic benefit. $w_{v}$ represents the weight assigned to the vth indicator. Considering that grain yield, WUE, and economic benefit were all core objectives of water–fertilizer optimization in this study and were considered equally important, equal weights were assigned to the three indicators, i.e., $w_{v}=1/3$. $Z_{v}^{best}$ and $Z_{v}^{worst}$ denote the positive and negative ideal solutions for the vth indicator, respectively. $D_{m}^{+}$ and $D_{m}^{-}$ represent the Euclidean distances between the mth water–fertilizer treatment and the positive and negative ideal solutions, respectively. $C_{m}$ is the relative closeness coefficient of the mth treatment. A larger $C_{m}$ value indicates that the corresponding treatment is closer to the comprehensive ideal state characterized by high grain yield, high WUE, and high economic benefit. TOPSIS ranking was performed separately for the two growing seasons, and representative optimal water–fertilizer treatments were selected based on the ranking stability across years.

#### 2.3.10. Data Analysis

Experimental data were processed and subjected to statistical analysis using Microsoft Excel 2024 and IBM SPSS Statistics 27. All results are presented as the mean of three replicates. Significant differences among treatments were evaluated using the least significant difference (LSD) test at the 0.05, 0.01, and 0.001 significance levels, indicated by *, **, and ***, respectively. Grain yield, water use efficiency, and economic benefit were selected as the key indicators for irrigation and fertilization optimization in winter wheat. Response surface methodology (RSM) and TOPSIS-based decision analysis were conducted in MATLAB R2024b, and all figures were generated using Origin 2024b.

## 3. Results

### 3.1. Canopy Development, Dry Matter Accumulation, and Crop Water Consumption Characteristics Under Water and Fertilizer Regulation

**Figure 4:**
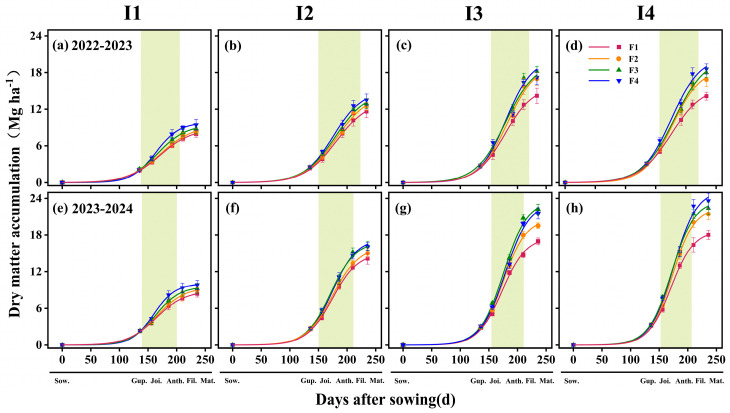
Dynamics of aboveground dry matter accumulation of winter wheat under different irrigation and fertilization treatments in 2022–2023 (**a**–**d**) and 2023–2024 (**e**–**h**). I1–I4 represent 60%, 80%, 100%, and 120% *ET_c_*, respectively; F1–F4 represent 70–65–25, 140–130–50, 210–195–75, and 280–260–100 kg ha^−1^ N–P_2_O_5_–K_2_O, respectively. Gup., Joi., Anth., Fil., and Mat. indicate green-up, jointing, anthesis, grain filling, and maturity, respectively. Values are means of three replicates, and error bars indicate standard errors.

**Figure 5:**
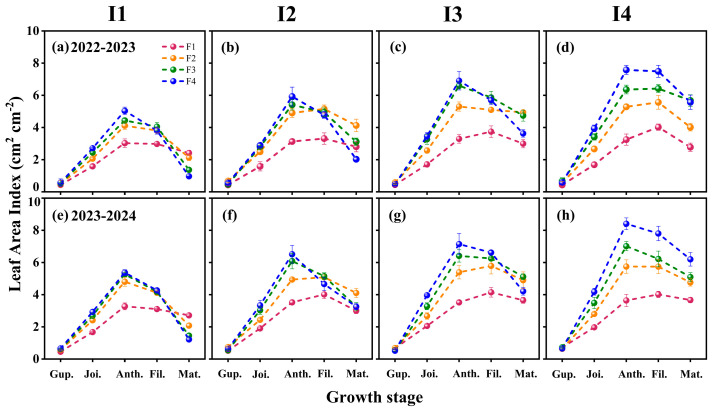
Dynamic changes in leaf area index (LAI) of winter wheat under different irrigation and fertilization treatments during 2022–2023 (**a**–**d**) and 2023–2024 (**e**–**h**). I1–I4 represent 60%, 80%, 100%, and 120% *ET_c_*, respectively; F1–F4 represent 70–65–25, 140–130–50, 210–195–75, and 280–260–100 kg ha^−1^ N–P_2_O_5_–K_2_O, respectively. Gup., Joi., Anth., Fil., and Mat. indicate green-up, jointing, anthesis, grain filling, and maturity, respectively. Values are means of three replicates, and error bars indicate standard errors.

As shown in Figure 4 and Figure 5, aboveground dry matter accumulation (DM) and leaf area index (LAI) of winter wheat exhibited distinct stage-dependent dynamics in both growing seasons. DM increased slowly from the green-up to jointing stages, followed by a rapid increase from jointing to grain filling, and then increased at a slower rate from grain filling to maturity, gradually reaching its maximum value. The seasonal pattern of LAI was generally consistent with that of DM, increasing rapidly after jointing and reaching a peak between anthesis and grain filling, before declining with leaf senescence. These results indicate that the period from jointing to grain filling is a critical phase for canopy expansion and biomass formation in winter wheat.

Irrigation and fertilization significantly affected DM at maturity, with a significant interaction between the two factors (Table 2). The combined ANOVA across the two growing seasons further confirmed significant effects of year, irrigation, fertilization, and irrigation × year on dry matter accumulation and crop evapotranspiration (Table S1). The highest values in both growing seasons were observed under the I4F4 treatment, reaching 18.60 and 23.60 Mg ha^−1^, respectively. DM increased markedly as irrigation increased from I1 to I3; however, the increment became smaller when irrigation was further increased from I3 to I4. The positive effect of fertilization on DM was also constrained by irrigation level. Under low irrigation conditions, additional fertilizer input resulted in only limited biomass gains, whereas under higher irrigation levels, fertilization had a more pronounced promotive effect on DM. The response pattern of LAI was similar to that of DM, with the highest values also recorded under I4F4, reaching 7.58 and 8.41 in the two growing seasons, respectively. Overall, higher water and fertilizer inputs enlarged canopy size and promoted biomass accumulation, but the associated gains showed diminishing marginal returns under high-input conditions.

As shown in Table 2, crop evapotranspiration (*ET*) responded significantly to water–fertilizer coupling, with irrigation exerting a more pronounced effect. Across treatments, *ET* ranged from 333.33 to 586.60 mm in 2022–2023 and from 386.55 to 653.14 mm in 2023–2024, with the highest values observed under I4F4 in both growing seasons. At the same fertilization level, *ET* increased continuously with increasing irrigation amount; at the same irrigation level, increased fertilization also generally enhanced *ET*, although the magnitude of increase was relatively small under low-irrigation conditions. These results indicate that water supply was the primary factor driving seasonal water consumption, whereas fertilization further enhanced crop water use mainly by promoting canopy expansion and biomass formation. Taken together, the changes in dry matter accumulation, LAI, and *ET* suggest that increasing water and fertilizer inputs can simultaneously promote canopy establishment and water consumption. However, under high water and fertilizer inputs, biomass gains tended to level off, whereas *ET* remained at a high level.

### 3.2. Responses of Chlorophyll Content and Leaf Gas Exchange to Water and Fertilizer Regulation

**Figure 6:**
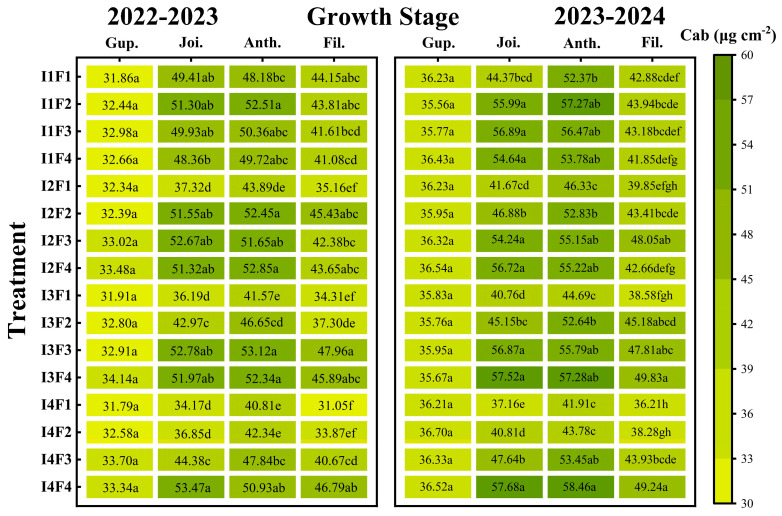
Heatmap of leaf chlorophyll content (*C_ab_*) of winter wheat under different irrigation and fertilization treatments during the 2022–2023 and 2023–2024 growing seasons. I1–I4 represent 60%, 80%, 100%, and 120% *ET_c_*, respectively; F1–F4 represent 70–65–25, 140–130–50, 210–195–75, and 280–260–100 kg ha^−1^ N–P_2_O_5_–K_2_O, respectively. Gup., Joi., Anth., and Fil. indicate green-up, jointing, anthesis, and grain filling, respectively. Values are means of three replicates; different lowercase letters indicate significant differences within the same growth stage and year at *p* < 0.05.

As shown in Figure 6, leaf chlorophyll content (*C_ab_*) in winter wheat generally followed an increasing-then-decreasing pattern over the growing season, with relatively high values mainly occurring from jointing to anthesis. Under the same irrigation level (I4), increased fertilization significantly enhanced *C_ab_* at anthesis, with I4F4 exceeding I4F1 by 24.8% and 39.49% in the two growing seasons, respectively. In contrast, under the low-fertilization treatment (F1), increasing irrigation alone did not maintain a high *C_ab_* level; compared with I1F1, *C_ab_* under I4F1 instead decreased by 15.27% and 19.93%, respectively. These results indicate that *C_ab_* depended more on coordinated water and nutrient supply than on continuous increases in either input alone.

Leaf net photosynthetic rate (*Pn*) responded more markedly to water–fertilizer treatments during the reproductive growth stage, with treatment differences mainly occurring at anthesis and grain filling (Table 3). The combined ANOVA including year effects for Pn and Tr is provided in Tables S2 and S3. Overall, higher *Pn* values were primarily observed under I3F3 and adjacent moderate-to-high water and fertilizer input treatments. In 2022–2023, *Pn* under I3F3 reached 23.33 and 20.88 μmol m^−2^ s^−1^ at anthesis and grain filling, respectively. Although the highest values in 2023–2024 were observed under I4F4, reaching 24.72 and 22.58 μmol m^−2^ s^−1^, respectively, I3F3 still maintained a relatively high assimilation capacity. At the same fertilization level of F3, increasing irrigation from I3 to I4 reduced *Pn* at anthesis and grain filling by 10.9–11.9% and 6.6–8.2% across the two growing seasons, respectively. These results suggest that further increasing irrigation beyond an appropriate water supply level does not continuously enhance leaf net photosynthetic rate.

In contrast to *Pn*, transpiration rate (*Tr*) responded more directly to water supply (Table 4). In both growing seasons, the highest *Tr* values at anthesis were observed under I4F1, reaching 5.97 and 6.28 mmol m^−2^ s^−1^, respectively. Compared with I3F3, *Tr* under I4F1 at anthesis increased by 16.2% and 24.1% in the two seasons, whereas *Pn* decreased by 32.1% and 44.2%, respectively. This indicates that enhanced transpiration under high-irrigation and low-fertilization conditions was not synchronously translated into improved photosynthetic assimilation. Overall, appropriate water–fertilizer coordination helped maintain higher leaf chlorophyll content and net photosynthetic rate; however, under higher irrigation levels, the increase in transpiration rate may exceed the improvement in photosynthetic rate, leading to a decoupling between leaf assimilation and transpiration.

### 3.3. Coupling Relationship Between Crop Water Consumption and Leaf Gas Exchange and the Thresholds of Luxury Transpiration

**Figure 7:**
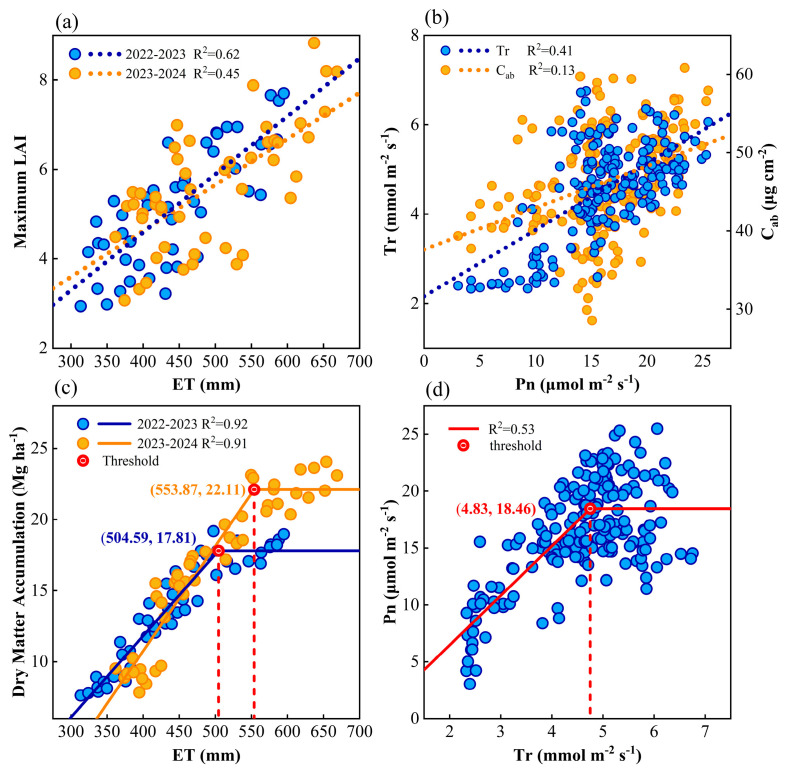
Coupling relationships between crop water consumption and leaf gas exchange, and threshold identification of luxury transpiration in winter wheat. (**a**) Relationship between seasonal evapotranspiration (*ET*) and maximum leaf area index (LAImax). (**b**) Relationships of net photosynthetic rate (*Pn*) with transpiration rate (*Tr*) and leaf chlorophyll content (*C_ab_*). (**c**) Linear plateau relationship between *ET* and dry matter accumulation (DM) at maturity. (**d**) Linear plateau relationship between *Tr* and *Pn*. Red dashed lines indicate threshold values.

As shown in Figure 7a, seasonal evapotranspiration (*ET*) was significantly positively correlated with the maximum leaf area index (LAI_max_), with coefficients of determination of 0.62 and 0.45 in the two growing seasons, respectively. This indicates that increases in LAI_max_ expanded the canopy transpiring surface and were accompanied by greater crop water consumption. However, the promotive effect of *ET* on dry matter accumulation (DM) did not continue indefinitely. As shown in Figure 7c, *ET* and DM at maturity exhibited a significant linear plateau relationship, with *ET* breakpoints of 504.59 and 553.87 mm in the 2022–2023 and 2023–2024 growing seasons, respectively, corresponding to DM plateau values of 17.81 and 22.11 Mg ha^−1^. Combined with Table 2, the thresholds in both years were located around the I3 irrigation level, specifically between I3F3 and I3F4 in the first season and between I3F2 and I3F3 in the second season. In both years, *ET* under the I3F3 treatment was only 1.71% below and 2.57% above the threshold, respectively, while DM had already reached 18.17 and 22.20 Mg ha^−1^, which were close to the corresponding plateau values. In contrast, although I4F4 maintained a larger canopy and higher water consumption, the increase in DM was very limited. These results indicate that once crop water consumption exceeded the level around I3F3, additional water input could no longer effectively promote dry matter accumulation.

The leaf-level results further supported the pattern observed at the crop-stand scale. As shown in Figure 7b, during the anthesis-to-grain-filling period, the correlation between *Pn* and *Tr* (R^2^ = 0.41) was markedly stronger than that between *Pn* and *C_ab_* (R^2^ = 0.13), indicating that variation in *Pn* was more closely coupled with transpiration than with chlorophyll content. As shown in Figure 7d, the relationship between *Tr* and *Pn* also followed a significant inear plateau pattern, with a breakpoint at *Tr* = 4.83 mmol m^−2^ s^−1^, corresponding to a *Pn* of 18.46 μmol m^−2^ s^−1^. Treatments near this threshold were mainly concentrated in the I3F3–I3F4 range, among which I3F3 can be regarded as a representative high-efficiency treatment close to the threshold. During anthesis and grain filling, *Tr* under I3F3 remained within 4.64–5.14 mmol m^−2^ s^−1^, while *Pn* was maintained at a relatively high level of 20.88–23.33 μmol m^−2^ s^−1^, and instantaneous photosynthetic water use efficiency (PWUE) ranged from 4.48 to 4.59 (Table 5). The combined ANOVA including year effects for PWUE is provided in Table S4. This indicates that, under this water–fertilizer combination, leaf transpiration and net photosynthetic assimilation remained well coordinated. By contrast, when irrigation increased from I3F3 to I4F3, *Tr* increased by 5.5–10.5%, whereas *Pn* decreased by 6.6–11.8%, accompanied by a 13.5–17.2% decline in PWUE. These results indicate that the additional water input was expressed mainly as enhanced transpiration rather than a corresponding increase in net photosynthesis. Taken together, both the crop-stand scale *ET*–DM relationship and the leaf-scale *Tr*–*Pn* relationship indicate that when water and fertilizer inputs exceeded the range around I3F3, the additional water consumption was expressed primarily as non-productive transpiration, that is, luxury transpiration occurred.

### 3.4. Effects of Irrigation–Fertilization Interaction on Grain Yield, Water Use Efficiency, and Economic Benefit

**Figure 8:**
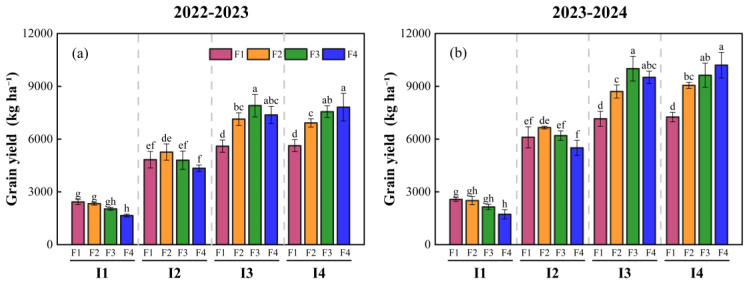
Effects of different irrigation and fertilization treatments on grain yield of winter wheat: (**a**) 2022–2023 growing season; (**b**) 2023–2024 growing season. I1, I2, I3, and I4 represent irrigation levels of 60%, 80%, 100%, and 120% *ET_c_*, respectively; F1, F2, F3, and F4 represent different fertilization levels. Error bars indicate standard deviation of three replicates. Different lowercase letters indicate significant differences among treatments within the same growing season at *p* < 0.05.

Winter wheat grain yield increased significantly with increasing irrigation level in both growing seasons, but the yield-promoting effect weakened under high-input conditions (Figure 8). In 2022–2023, the mean grain yield reached its maximum at the I3 level, with a value of 7002.7 kg ha^−1^, representing increases of 231.6% and 45.6% compared with I1 and I2, respectively, and only 0.3% higher than I4. In 2023–2024, the highest yield was observed at I4, reaching 9034.4 kg ha^−1^, but this was only 2.2% higher than that at I3. The yield response to fertilization was strongly constrained by water availability. Under I1 and I2, increasing fertilizer input did not consistently enhance grain yield, whereas under I3 and I4, both F3 and F4 maintained relatively high yields; however, the yield increment from F3 to F4 was markedly reduced. These results indicate that moderate water–fertilizer coupling is conducive to yield formation, whereas further increases in input levels are unlikely to produce sustained and significant yield gains.

**Figure 9:**
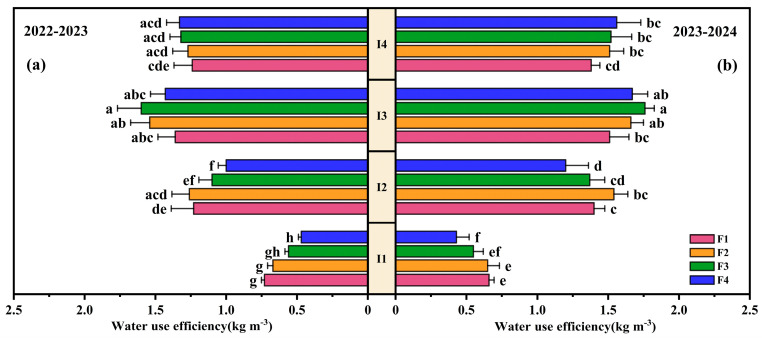
Effects of different irrigation and fertilization treatments on water use efficiency of winter wheat: (**a**) 2022–2023 growing season; (**b**) 2023–2024 growing season. I1–I4 indicate irrigation levels of 60%, 80%, 100%, and 120% *ET_c_*, respectively; F1–F4 indicate different fertilization levels. Error bars represent the standard deviation of three replicates. Different lowercase letters indicate significant differences among treatments within the same growing season at *p* < 0.05.

Winter wheat WUE ranged from 0.47 to 1.59 kg m^−3^ and from 0.43 to 1.76 kg m^−3^ in the two growing seasons, respectively (Figure 9). Overall, under the low irrigation levels of I1 and I2, water deficit constrained the beneficial effects of fertilization, and increased fertilizer input could not be effectively converted into yield advantages. In some high-fertilizer treatments, WUE even declined due to increased water consumption. When irrigation increased to I3, water availability was improved and WUE reached its highest level. In particular, I3F3 achieved the highest WUE in both seasons, with values of 1.59 and 1.76 kg m^−3^, respectively. This indicates that moderate fertilization under sufficient but non-excessive water supply was most conducive to maximizing WUE. However, when irrigation was further increased to I4, WUE did not improve further but decreased by 12.9% and 9.5% in the two seasons, respectively, suggesting that the additional water input mainly increased water consumption rather than producing a synchronous yield increase.

Table 6 shows that irrigation, fertilization, and their interaction significantly affected the output value and net return of winter wheat (*p* < 0.01). The combined ANOVA across the two growing seasons further showed that year, irrigation, fertilization, I × F, and I × Y significantly affected output value and economic benefits, whereas F × Y and I × F × Y were not significant (Table S5). Net return ranged from −7222.76 to 7974.05 CNY ha^−1^ in 2022–2023 and from −7097.85 to 12,966.42 CNY ha^−1^ in 2023–2024. The average net return under I1 was negative in both seasons, indicating that water deficiency severely constrained economic performance. When irrigation increased from I2 to I3, the average net return increased markedly, by 328.14% and 135.14% in the two seasons, respectively. However, further increasing irrigation to I4 did not improve net return, suggesting that the economic benefit of irrigation tended to saturate beyond I3. In terms of fertilization, excessive fertilizer input under low-irrigation conditions reduced net return due to increased input costs. Under I3, F3 achieved the highest net return in both seasons, reaching 7974.05 and 12,966.42 CNY ha^−1^, respectively. Nevertheless, when fertilization increased from F3 to F4, net return decreased by 28.8% and 17.13% in the two seasons, respectively, indicating that a moderate water–fertilizer combination was more conducive to maximizing economic benefits.

### 3.5. Identification of a Cross-Year Robust Water–Fertilizer Management Range Under Multi-Objective Optimization

To identify a water–fertilizer management range with high applicability across different growing seasons, quadratic response surface models were developed for grain yield, water use efficiency (WUE), and economic benefit as functions of irrigation amount and fertilizer input, using data from the two-year winter wheat field experiment (Table 7). The results showed that all models for each growing season were highly significant (*p* < 0.001), with coefficients of determination (R^2^) greater than 0.95. Moreover, the differences between adjusted R^2^ and R^2^ were less than 0.03, indicating good model performance and suggesting that the models could accurately characterize the response patterns of winter wheat under different water and fertilizer conditions.

**Figure 10:**
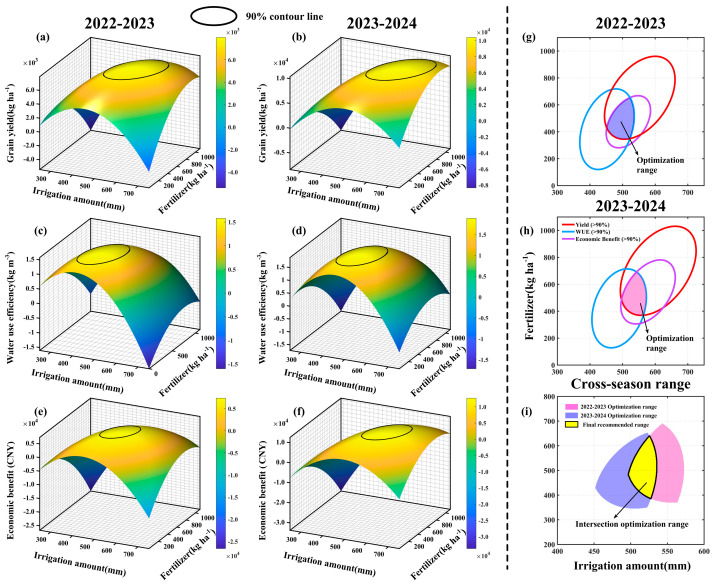
Three-dimensional response surfaces and spatial overlay analysis for identifying a robust irrigation-fertilization management strategy. (**a**–**f**) Interactive effects of irrigation and fertilization on grain yield, water use efficiency (WUE), and economic benefit during the 2022–2023 and 2023–2024 growing seasons. (**g**,**h**) Confidence intervals satisfying more than 90% of the three objectives in the two growing seasons. (**i**) Final recommended cross-season overlapping optimization region.

The three-dimensional response surface plots (Figure 10) visually illustrate the interactive effects of water–fertilizer coupling on the evaluated variables. All response surfaces exhibited a downward-opening convex shape, indicating a nonlinear trend in which the response variables initially increased and then declined with increasing water and fertilizer inputs, thereby suggesting an inhibitory effect of excessive inputs. To further identify a water–fertilizer management range that could simultaneously achieve high yield, high efficiency, and high economic return, 90% of the predicted maximum value of each model in each growing season was defined as the high-performance threshold. Spatial overlay analysis was then used to screen water–fertilizer combinations that simultaneously satisfied the high-performance requirements for grain yield, WUE, and net economic benefit. The results showed that the integrated high-performance feasible domain in the 2022–2023 growing season was 461.72–545.09 mm for irrigation and 345.49–628.33 kg ha^−1^ for fertilizer input, whereas that in the 2023–2024 growing season was 506.21–588.78 mm for irrigation and 369.54–726.65 kg ha^−1^ for fertilizer input. These high-performance domains were mainly located around the I3F3 treatment and its adjacent water–fertilizer range, indicating that moderate-to-high irrigation and fertilization levels were more favorable than excessive inputs for achieving balanced yield, WUE, and economic return. Although the high-performance ranges shifted to some extent between the two seasons due to interannual climatic variability, a clear overlapping region was still identified. To account for interannual climatic variation and ensure the applicability of the management strategy, the intersection of the two high-performance feasible domains was defined as the robust interannual water–fertilizer management range, with irrigation amounts of 506.21–545.09 mm and fertilizer inputs of 369.54–628.33 kg ha^−1^.

### 3.6. TOPSIS-Based Comprehensive Evaluation of Water–Fertilizer Treatments

The TOPSIS-based comprehensive evaluation using grain yield, WUE, and net economic benefit showed that I3F3 achieved the highest overall evaluation score in both growing seasons, with relative closeness coefficients ($C_{m}$) of 1.000 and 0.990, respectively. This indicates that I3F3 was closest to the comprehensive ideal state characterized by high yield, high WUE, and high economic benefit. In 2022–2023, I3F3, I3F2, and I4F3 ranked as the top three treatments, with $C_{m}$ values of 1.000, 0.936, and 0.886, respectively. In 2023–2024, I3F3, I3F4, and I3F2 ranked as the top three treatments, with $C_{m}$ values of 0.990, 0.888, and 0.883, respectively (Table 8). Overall, the treatments with superior comprehensive performance across the two years were mainly concentrated around I3F3 and its adjacent water–fertilizer levels, further indicating that moderate-to-high water and fertilizer inputs were more conducive to the coordinated improvement of grain yield, WUE, and economic benefits than the highest input levels.

Integrating the TOPSIS ranking, the high-performance ranges derived from response surface analysis, and the luxury transpiration thresholds, I3F3 can be identified as the representative optimal treatment under the present experimental conditions, effectively balancing high yield, high WUE, and high economic benefits. This treatment was located near the linear plateau thresholds of the *ET*–DM and *Tr*–*Pn* relationships and was adjacent to the overlapping high-performance response surface region identified across the two years. These findings indicate that maintaining water and fertilizer inputs at levels close to I3F3 can help reduce the risk of luxury transpiration and inefficient water use associated with further increases in irrigation and fertilization, thereby facilitating the coordinated improvement of winter wheat yield, WUE, and economic benefits.

## 4. Discussion

### 4.1. Synergistic Regulation of Water and Fertilizer Promotes Efficient Canopy Establishment and Maintains Leaf Photosynthetic Function

Appropriate irrigation can maintain leaf turgor and cell expansion, promote leaf area development and canopy closure, and, to some extent, prolong leaf functional duration. Meanwhile, rational fertilization facilitates chlorophyll synthesis, maintains photosynthetic enzyme activity, and enhances the effective utilization of light energy by leaves, thereby improving leaf photosynthetic performance [35,36,37]. The results of the two-year field experiment in the present study were generally consistent with these findings. When water and fertilizer inputs increased from low levels to I3F3 and its adjacent levels, leaf area index (LAI), dry matter accumulation (DM), leaf chlorophyll content (*C_ab_*), and net photosynthetic rate (*Pn*) of winter wheat during the jointing to grain-filling stages were markedly improved. This indicates that appropriate water and nutrient supply can effectively alleviate water and nutrient limitations during winter wheat growth in the arid region of southern Xinjiang, thereby promoting leaf area expansion, canopy light interception, and assimilate accumulation. However, with further increases in water and fertilizer inputs, the increments in dry matter accumulation, leaf area index, and leaf photosynthetic performance tended to diminish, and some indicators even declined. Previous studies have also reported that although winter wheat growth can be improved by increasing irrigation and fertilization, the optimal combinations often occur under moderate or moderately high input levels rather than under the highest input treatment [38]. These findings suggest that the response of winter wheat to water and fertilizer inputs is not linear; once water and nutrient limitations are largely alleviated, further increases in inputs are unlikely to produce synchronous improvements in canopy establishment and leaf photosynthetic capacity.

Water and nutrients do not act in isolation, but jointly regulate crop physiological growth through complex synergistic interactions [39,40]. To some extent, water availability determines the threshold for crop uptake and utilization of nutrient inputs [41]. In the present study, under excessive irrigation (I4), increasing fertilizer input from F1 to F4 substantially enhanced biomass accumulation, whereas under low irrigation (I1), the promotive effect of the same increase in fertilizer input was relatively limited. This may be attributed to water deficit restricting the dissolution and transport of soil nutrients, making higher nutrient inputs difficult to translate effectively into crop growth advantages, and potentially further inhibiting root water uptake and plant metabolism [42,43,44]. Conversely, under nutrient-limited conditions, increasing irrigation alone may promote vegetative growth and canopy expansion, but it is unlikely to sustain high chlorophyll content and photosynthetic assimilation capacity [36,45]. For example, *C_ab_* and *Pn* under I4F1 at anthesis were lower than those under I1F1, possibly because sufficient irrigation promoted rapid shoot biomass accumulation and leaf area expansion, whereas low nutrient supply led to nutrient dilution in leaves and constrained chlorophyll synthesis and photosynthetic enzyme activity [46,47]. Overall, the optimization of canopy establishment and photosynthetic function in winter wheat depends not on increasing water or nutrient input alone, but on whether irrigation and fertilization are synchronously matched during key growth stages. This transition from synergistic enhancement to diminishing responses also provides a physiological basis for subsequently identifying luxury transpiration thresholds from the *ET*–DM and *Tr*–*Pn* relationships.

### 4.2. Crop-Stand and Leaf-Scale Thresholds Reveal the Mechanisms Underlying Luxury Transpiration

High water and fertilizer inputs did not continuously enhance effective assimilation in winter wheat; instead, they were more likely to induce luxury transpiration and non-productive water consumption. The present study showed that, with increasing water and fertilizer inputs, crop evapotranspiration (*ET*) at the crop-stand scale continued to increase with canopy expansion, whereas its positive effect on DM did not increase indefinitely but shifted to a plateau after reaching a certain level. At the leaf scale, transpiration rate (*Tr*) and *Pn* similarly exhibited a linear plateau relationship, indicating that transpiration could continue to increase while the gain in photosynthetic assimilation had already diminished markedly. Under appropriate water and fertilizer conditions, additional resources were mainly used to promote canopy establishment and carbon assimilation. However, once inputs exceeded the optimal range, the dominant constraint shifted from insufficient resource supply to reduced resource use efficiency, with additional inputs contributing more to increased water consumption and lower assimilation efficiency than to sustained and effective biomass accumulation.

At the crop-stand scale, the formation of luxury transpiration under high water and fertilizer inputs is first manifested as a faster increase in water consumption than in dry matter accumulation following excessive canopy expansion. Previous studies have shown that irrigation and fertilization can simultaneously increase LAI and DM, but once inputs exceed the appropriate range, the marginal benefits for dry matter accumulation and grain yield decline markedly [48]. It has also been reported that an overly luxuriant canopy can intensify within-canopy shading, reducing the contribution of newly developed leaf area to light interception and assimilation, whereas canopy maintenance costs and evapotranspiration may continue to increase [49]. These findings are consistent with the present results, in which *ET* continued to increase while DM tended to plateau. In addition, high irrigation frequency and excessive irrigation amounts can keep the surface soil frequently moist, particularly during the early growth stages when the canopy has not yet fully closed, thereby substantially increasing the proportion of soil evaporation from the exposed interplant surface and further enhancing non-productive water loss [50,51]. Therefore, luxury transpiration at the crop-stand scale is not merely the result of enhanced crop transpiration, but also reflects inefficient water consumption jointly driven by excessive canopy growth and increased soil evaporation.

At the leaf scale, the asynchronous enhancement of transpiration and assimilation under high water and fertilizer inputs represents another key mechanism underlying luxury transpiration. Previous studies have shown that, with improved water supply, stomatal opening first and directly promotes transpiration, whereas *Pn* is regulated not only by stomatal behavior but also by non-stomatal factors, including chloroplast biochemical capacity and leaf nitrogen status. Consequently, *Pn* often reaches a plateau earlier than *Tr* [52]. Increased fertilization has also been reported to enhance leaf carboxylation rate and Rubisco activity, thereby helping maintain relatively high Pn within an appropriate nutrient supply range [53]. However, when water and nutrient supply exceeds the current photosynthetic capacity of leaves or the growth demand of plants, the promotive effect of stomatal opening on water loss persists, whereas *Pn* and instantaneous water use efficiency are difficult to improve synchronously. This indicates that additional water is mainly converted into transpiration consumption rather than further improvement in net photosynthetic assimilation.

### 4.3. From Synergistic Gains to Objective Trade-Offs: Integrated Advantages of the I3F3-Adjacent Range

The I3F3-adjacent range was not an incidental optimum for a single indicator, but rather a critical threshold zone in which the response of winter wheat to water and fertilizer inputs shifted from synergistic gains to objective trade-offs. Under low water and fertilizer inputs, insufficient water and nutrient supply jointly constrained canopy establishment, photosynthetic assimilation, and grain yield formation. Therefore, increasing irrigation and fertilization effectively alleviated resource limitations and promoted the synchronous improvement of grain yield, WUE, and economic benefits. However, when water and fertilizer inputs increased to the range adjacent to I3F3, the major resource limitations had been largely relieved, and canopy size, leaf photosynthetic capacity, and dry matter accumulation gradually approached an efficient production state. Within this range, additional water and nutrient inputs could still be effectively converted into assimilates and economic output, thereby maintaining a high level of coordination among grain yield, WUE, and net return. The TOPSIS-based comprehensive evaluation, the identified *ET*–DM and *Tr*–*Pn* thresholds, and the response surface-derived high-performance ranges all converged within this region, indicating that the I3F3-adjacent range represents not only an efficient coupling zone between crop water consumption and leaf photosynthetic processes, but also a robust water–fertilizer management range for the coordinated optimization of grain yield, WUE, and economic benefits across years.

This advantage essentially arose because the I3F3-adjacent range remained close to the threshold for efficient water and fertilizer utilization, whereas higher input levels gradually entered a stage of diminishing marginal returns. As resource supply continued to increase, the dominant constraints on further yield improvement may no longer have been water and nutrient deficiencies but instead shifted toward internal limitations such as canopy structural optimization, sink capacity, and assimilate translocation efficiency [54]. Previous studies have shown that spike formation and sink capacity expansion are stage-dependent and inherently limited; therefore, even if higher photosynthetic assimilation is maintained by increasing water and fertilizer supply at later stages, the additional assimilates cannot be continuously converted into grain yield at a proportional rate [55,56,57]. Under these conditions, further increases in irrigation and fertilizer inputs are expressed more as increases in water consumption and input costs than as equivalent gains in yield and economic benefits. Meanwhile, excessive water supply is more likely to induce luxury transpiration, whereby transpiration rate and crop-stand water consumption continue to increase, while net photosynthetic rate and dry matter accumulation gradually approach a plateau, thereby reducing the yield return per unit of water consumed. The combined effects of weakened marginal yield gains, reduced WUE, and increased input costs ultimately diminished the overall benefits of high water and fertilizer treatments. Therefore, the I3F3-adjacent range should not be interpreted as a conservative “compromise point”, but rather as an efficiency-optimal zone characterized by high resource use efficiency, limited intensification of luxury transpiration, and sustained marginal benefit advantages.

### 4.4. Robust Water–Fertilizer Optimization for Winter Wheat Production in Arid Regions: Implications, Limitations, and Future Perspectives

The significance of multi-objective optimization in agricultural production lies not only in reconciling the trade-offs among grain yield, WUE, and economic benefits within a given year, but also in identifying robust management strategies that can adapt to interannual climatic variability [58]. For winter wheat production in the extremely arid oasis regions of southern Xinjiang, variations in precipitation patterns and evaporative demand can substantially alter crop water consumption, resource use efficiency, and economic benefits, thereby shifting the response surfaces of grain yield, WUE, and economic benefits across years. As a result, fixed single-point water–fertilizer schemes may be difficult to apply stably over the long term [59,60]. Previous studies on water–fertilizer optimization have often established unified response surface models by pooling multi-year data. Although this approach can provide an average water–fertilizer response relationship, it may smooth interannual differences and weaken the characterization of shifts in optimal water–fertilizer ranges under different climatic conditions, thereby reducing the robustness and practical interpretability of the recommended management strategies [61]. Accordingly, in the present study, response surface models describing the effects of irrigation amount and fertilizer input on grain yield, WUE, and net economic benefit were constructed separately for the two growing seasons. In each year, 90% of the predicted maximum value for each indicator was defined as the high-performance threshold, and the overlapping region that simultaneously satisfied the requirements for high yield, high WUE, and high economic benefit in both years was further extracted. This strategy emphasizes not the theoretical optimum in a single year, but an efficient water–fertilizer range capable of maintaining high production performance under different annual conditions. Therefore, the robust range identified in this study better reflects the adaptability of water–fertilizer management to interannual climatic variability and is more suitable as a field reference range for precise water and fertilizer management of winter wheat in the arid regions of southern Xinjiang.

This study proposed a robust water–fertilizer management range for winter wheat production in the arid region of southern Xinjiang based on two consecutive years of field experiments, providing a theoretical basis for shifting from empirically based high-input management toward precise water–fertilizer regulation in arid agricultural systems. Nevertheless, future studies could further extend this work from three perspectives: spatiotemporal scale, environmental effects, and process mechanisms. First, although the two-year experiment captured certain interannual meteorological differences, long-term climatic variability, extreme heat events, episodic drought, and differences in soil texture and cultivar type may further affect the responses of winter wheat to water and fertilizer inputs. Therefore, validation across multiple sites, years, and cultivars is needed to improve the regional applicability and extension reliability of the recommended management range. Second, oasis agriculture in southern Xinjiang has long faced challenges related to water scarcity, soil salinity accumulation, and nutrient residues. Future evaluations could incorporate environmental indicators such as salt transport, nitrate-N residue, fertilizer use efficiency, and leaching risk, in addition to grain yield, WUE, and economic benefits, to develop an integrated optimization framework that balances production benefits with ecological risk control. Meanwhile, process-based crop models such as DSSAT and AquaCrop, together with canopy remote sensing techniques, could be used to conduct long-term scenario simulations and regional-scale extrapolation of the optimized water–fertilizer range, thereby developing more adaptive and operational strategies for precise irrigation and fertilization management of winter wheat in arid regions [62,63].

## 5. Conclusions

Based on a two-year field experiment with irrigation and fertilization gradients in the extremely arid oasis region of southern Xinjiang, this study systematically analyzed the effects of coordinated water–fertilizer management on canopy development, leaf gas exchange, evapotranspiration, grain yield, water use efficiency (WUE), and economic benefits of winter wheat. Linear plateau models, response surface analysis, and TOPSIS evaluation were used to identify luxury transpiration thresholds and robust interannual high-performance water–fertilizer ranges. The results showed that: (1) Appropriate water and nutrient supply significantly promoted leaf area expansion and canopy establishment, maintained leaf chlorophyll content and photosynthetic capacity, and enhanced dry matter accumulation, thereby improving grain yield, WUE, and economic benefits. (2) When water and fertilizer inputs exceeded the optimal range, both the crop-stand-scale ET–DM relationship and the leaf-scale *Tr*–*Pn* relationship exhibited distinct linear plateau patterns. Seasonal ET thresholds were 504.59 mm and 553.87 mm, and the leaf-scale Tr threshold was 4.83 mmol m^−2^ s^−1^, mainly located around I3F3 and its adjacent range. Beyond these thresholds, additional water consumption primarily manifested as luxury transpiration. (3) Separate response surface analyses for the two growing seasons combined with TOPSIS evaluation indicated that the I3F3-adjacent range maintained high yield, WUE, and economic benefits, with I3F3 achieving the highest overall performance in both seasons. (4) Based on the intersection of two-year high-performance regions, a robust interannual feasible range was identified, with irrigation amounts of 506.21–545.09 mm and total N–P_2_O_5_–K_2_O inputs of 369.54–628.33 kg ha^−1^. Overall, regulating water and fertilizer inputs within the I3F3-adjacent range can reduce non-productive water consumption and luxury transpiration risk while achieving coordinated optimization of yield, WUE, and economic benefits in winter wheat.

## Figures and Tables

**Table 1:** Physicochemical properties of soil at 0–30 cm depth.

Soil Properties	Value
pH	8.15
Organic matter (g kg^−1^)	3.78
Available phosphorus (mg kg^−1^)	9.84
Available potassium (mg kg^−1^)	172.47
Alkaline hydrolyzable nitrogen (mg kg^−1^)	3.11

**Table 2:** Effects of different irrigation and fertilization treatments on dry matter accumulation at maturity and Crop evapotranspiration of winter wheat.

Treatment		Dry Matter Accumulation at Maturity (kg ha^−1^)		Crop Evapotranspiration (mm)	
		2022–2023	2023–2024	2022–2023	2023–2024
I1	F1	7986.02 g	8389.91 h	333.33 j	391.06 g
	F2	8338.90 fg	9034.42 h	348.36 j	386.55 g
	F3	8808.15 fg	9259.77 h	363.81 ij	393.83 g
	F4	9460.40 f	9811.10 h	355.15 j	402.20 g
I2	F1	11,609.80 e	14,148.73 g	393.55 hi	434.66 f
	F2	12,440.40 de	15,005.60 fg	416.92 gh	430.86 f
	F3	12,936.29 cde	16,242.76 ef	436.28 fg	452.35 ef
	F4	13,530.36 cd	16,090.11 ef	434.62 fg	456.69 ef
I3	F1	14,207.00 c	16,968.98 de	411.70 gh	475.42 e
	F2	16,949.33 b	19,539.67 c	465.10 ef	523.37 d
	F3	18,172.96 ab	22,200.97 b	495.96 de	568.09 c
	F4	17,224.85 b	21,543.33 b	516.07 cd	569.89 c
I4	F1	14,151.93 c	18,024.21 cd	454.58 f	527.36 d
	F2	16,836.13 b	21,443.86 b	545.52 bc	599.14 b
	F3	17,993.63 ab	22,364.33 ab	574.90 ab	633.08 a
	F4	18,604.67 a	23,600.32 a	586.60 a	653.14 a
I		***	***	***	***
F		***	***	***	***
I × F		**	**	**	***

Note: I1–I4 represent 60%, 80%, 100%, and 120% *ET_c_*, respectively; F1–F4 represent 70–65–25, 140–130–50, 210–195–75, and 280–260–100 kg ha^−1^ N–P_2_O_5_–K_2_O, respectively. Values are means of three replicates. Different lowercase letters within the same column indicate significant differences at *p* < 0.05. ** and *** indicate significance at *p* < 0.01 and *p* < 0.001, respectively.

**Table 3:** Effects of different irrigation and fertilization treatments on leaf net photosynthetic rate (Pn) of winter wheat at different growth stages.

*Pn* (μmol m^−2^ s^−1^)			2022–2023				2023–2024		
Treatment		Green-Up	Jointing	Anthesis	Filling	Green-Up	Jointing	Anthesis	Filling
I1	F1	10.84 abc	17.18 cd	22.01 a	19.51 ab	11.57 ab	17.56 ef	20.38 bc	17.48 cde
	F2	10.60 abc	18.93 a	15.73 ef	10.28 f	10.77 ab	20.08 ab	13.08 f	12.53 f
	F3	10.39 abc	18.36 abc	15.28 efg	10.19 f	10.56 b	19.61 abc	12.61 f	8.68 g
	F4	10.02 c	17.54 bcd	13.94 g	6.22 g	11.15 ab	18.99 abcde	11.99 f	5.55 h
I2	F1	10.92 abc	14.30 e	17.42 cd	14.69 de	11.62 ab	15.52 gh	14.47 def	16.26 de
	F2	11.12 ab	18.35 abc	22.94 a	19.85 ab	11.58 ab	18.50 bcde	20.86 b	18.27 bcd
	F3	10.71 abc	18.42 abc	14.66 fg	10.45 f	11.28 ab	19.30 abcd	16.54 de	14.49 ef
	F4	10.30 bc	18.89 ab	15.65 ef	8.45 f	10.83 ab	19.89 ab	14.99 def	6.79 gh
I3	F1	11.00 abc	13.18 ef	16.48 de	13.87 e	11.78 a	14.36 h	13.93 ef	15.70 de
	F2	10.96 abc	16.77 d	20.09 b	18.09 bc	11.57 ab	16.86 fg	20.77 b	18.32 bcd
	F3	11.38 a	18.71 ab	23.33 a	20.88 a	11.80 a	18.15 cdef	23.19 ab	20.94 ab
	F4	10.98 abc	17.79 abcd	18.04 c	16.37 cd	11.16 ab	20.42 a	17.45 cd	16.34 de
I4	F1	10.67 abc	12.01 f	15.86 ef	13.95 e	11.60 ab	14.04 h	12.93 f	14.86 ef
	F2	10.83 abc	12.89 f	16.33 de	15.50 de	11.68 ab	14.83 h	13.74 ef	16.36 de
	F3	11.31 ab	18.12 abc	20.57 b	18.61 abc	11.88 a	17.97 def	21.30 b	19.56 bc
	F4	11.18 ab	19.09 a	22.11 a	19.02 ab	11.68 ab	17.47 ef	24.72 a	22.58 a
I		*	***	***	***	ns	***	***	**
F		ns	***	**	***	ns	**	***	***
I × F		ns	***	***	***	ns	**	***	**

Note: *Pn* represents leaf net photosynthetic rate. I1–I4 represent 60%, 80%, 100%, and 120% *ET_c_*, respectively; F1–F4 represent 70–65–25, 140–130–50, 210–195–75, and 280–260–100 kg ha^−1^ N–P_2_O_5_–K_2_O, respectively. Values are means of three replicates. Different lowercase letters within the same column indicate significant differences at *p* < 0.05. ns, *, **, and *** indicate no significance and significance at *p* < 0.05, *p* < 0.01, and *p* < 0.001, respectively.

**Table 4:** Effects of different irrigation and fertilization treatments on leaf transpiration rate (Tr) of winter wheat.

*Tr* (mmol m^−2^ s^−1^)			2022–2023				2023–2024		
Treatment		Green-Up	Jointing	Anthesis	Filling	Green-Up	Jointing	Anthesis	Filling
I1	F1	4.47 a	4.79 cd	4.95 de	4.59 cd	4.53 abc	4.79 cde	5.17 de	4.66 c
	F2	4.60 a	3.92 ef	4.32 f	3.06 f	4.16 bcd	4.59 de	4.00 i	3.39 d
	F3	4.28 ab	3.99 ef	4.08 f	2.72 fg	3.97 cd	4.41 e	4.04 hi	2.46 e
	F4	3.95 b	3.77 f	3.33 g	2.46 g	3.80 d	4.58 de	4.50 ghi	2.42 e
I2	F1	4.42 ab	5.64 a	5.48 bc	4.83 bc	4.77 ab	5.64 a	5.57 cd	5.05 ab
	F2	4.60 a	4.91 bcd	5.30 bcd	4.74 bcd	4.30 abcd	5.34 abc	5.16 de	4.58 c
	F3	4.57 a	3.85 ef	4.03 f	3.01 f	4.22 abcd	4.99 abcde	4.56 fgh	3.39 d
	F4	4.37 ab	3.88 ef	4.29 f	2.65 fg	4.22 abcd	4.33 e	4.33 ghi	2.47 e
I3	F1	4.54 a	5.36 abc	5.66 ab	5.09 ab	4.49 abc	5.22 abcd	5.98 abc	5.19 ab
	F2	4.37 ab	5.26 abc	5.36 bcd	4.66 bcd	4.33 abcd	5.60 a	6.18 ab	4.81 bc
	F3	4.72 a	5.09 abcd	5.14 cde	4.64 bcd	4.18 abcd	4.87 bcde	5.06 def	4.67 c
	F4	4.45 a	4.45 de	4.83 e	3.93 e	4.24 abcd	4.42 e	4.70 efg	3.66 d
I4	F1	4.36 ab	5.22 abc	5.97 a	5.37 a	4.26 abcd	5.39 abc	6.28 a	5.33 a
	F2	4.56 a	5.52 ab	5.70 ab	5.03 abc	4.82 a	5.51 ab	6.12 ab	5.08 ab
	F3	4.60 a	5.14 abc	5.43 bc	4.88 bc	4.48 abc	5.66 a	5.83 abc	4.96 abc
	F4	4.68 a	4.83 cd	5.11 cde	4.27 de	4.36 abcd	5.38 abc	5.70 bc	5.28 a
I		ns	***	***	***	*	***	***	***
F		ns	***	***	***	*	***	***	***
I × F		ns	***	*	***	ns	*	**	***

Note: *Tr* represents leaf transpiration rate. I1–I4 represent 60%, 80%, 100%, and 120% *ET_c_*, respectively; F1–F4 represent 70–65–25, 140–130–50, 210–195–75, and 280–260–100 kg ha^−1^ N–P_2_O_5_–K_2_O, respectively. Values are means of three replicates. Different lowercase letters within the same column indicate significant differences at *p* < 0.05. ns, *, **, and *** indicate no significance and significance at *p* < 0.05, *p* < 0.01, and *p* < 0.001, respectively.

**Table 5:** Leaf photosynthetic water use efficiency (PWUE) of winter wheat at the anthesis and grain-filling stages under different irrigation and fertilization treatments.

PWUE (μmol CO_2_ mmol^−1^ H_2_O)		2022–2023		2023–2024	
Treatment		Anthesis	Filling	Anthesis	Filling
I1	F1	4.46 a	4.26 ab	3.95 abc	3.77 abcd
	F2	3.64 cd	3.37 bcde	3.28 cdef	3.71 abcd
	F3	3.77 bc	3.77 abc	3.10 def	3.53 abcd
	F4	4.22 ab	2.52 e	2.64 efg	2.27 e
I2	F1	3.18 de	3.04 cde	2.61 fg	3.22 bcde
	F2	4.34 a	4.21 ab	4.05 abc	4.00 abc
	F3	3.63 cd	3.48 bcd	3.62 bcd	4.28 ab
	F4	3.65 cd	3.18 cde	3.46 cd	2.76 de
I3	F1	2.93 ef	2.75 de	2.33 g	3.03 cde
	F2	3.75 bc	3.90 abc	3.36 cde	3.82 abcd
	F3	4.54 a	4.50 a	4.59 a	4.48 a
	F4	3.74 bc	4.17 ab	3.74 bcd	4.47 a
I4	F1	2.66 f	2.62 de	2.06 g	2.78 de
	F2	2.87 ef	3.10 cde	2.26 g	3.23 bcde
	F3	3.80 bc	3.82 abc	3.66 bcd	3.95 abc
	F4	4.34 a	4.48 a	4.34 ab	4.28 ab
I		***	*	**	*
F		***	**	***	**
I × F		***	***	***	**

Note: PWUE represents leaf photosynthetic water use efficiency. I1–I4 represent 60%, 80%, 100%, and 120% *ET_c_*, respectively; F1–F4 represent 70–65–25, 140–130–50, 210–195–75, and 280–260–100 kg ha^−1^ N–P_2_O_5_–K_2_O, respectively. Values are means of three replicates. Different lowercase letters within the same column indicate significant differences at *p* < 0.05. I, F, and I × F indicate the effects of irrigation, fertilization, and their interaction, respectively. *, **, and *** indicate significance at *p* < 0.05, *p* < 0.01, and *p* < 0.001, respectively.

**Table 6:** Effects of different irrigation and fertilization treatments on the economic performance of winter wheat.

Treatment		Input Values of Consumable Items (CNY ha^−1^)					Output CNY ha^−1^)		Economic Benefit (CNY ha^−1^)	
		I_w_		I_f_	I_s_	I_o_				
		2022–2023	2023–2024				2022–2023	2023–2024	2022–2023	2023–2024
I1	F1	896.31	969.00	1050.12	3000	3000	5680.94 f	6063.88 f	−2265.50 fg	−1955.24 e
	F2	896.31	969.00	2100.24	3000	3000	5448.46 fg	5915.43 f	−3548.10 g	−3153.81 ef
	F3	896.31	969.00	3150.36	3000	3000	4761.16 fg	5064.16 f	−5285.51 h	−5055.20 fg
	F4	896.31	969.00	4200.48	3000	3000	3874.03 g	4071.64 f	−7222.76 i	−7097.85 g
I2	F1	1135.08	1232.01	1050.12	3000	3000	11,301.44 de	14,401.26 de	3116.24 d	6119.13 bc
	F2	1135.08	1232.01	2100.24	3000	3000	12,313.76 cd	15,699.73 cd	3078.44 d	6367.48 bc
	F3	1135.08	1232.01	3150.36	3000	3000	11,230.51 de	14,630.18 de	945.07 e	4247.81 c
	F4	1135.08	1232.01	4200.48	3000	3000	10,163.20 e	12,984.20 e	−1172.37 f	1551.71 d
I3	F1	1373.85	1495.02	1050.12	3000	3000	13,098.29 c	16,884.15 c	4674.32 cd	8339.01 b
	F2	1373.85	1495.02	2100.24	3000	3000	16,697.46 ab	20,543.45 b	7223.37 ab	10,948.18 a
	F3	1373.85	1495.02	3150.36	3000	3000	18,498.26 a	23,611.80 a	7974.05 a	12,966.42 a
	F4	1373.85	1495.02	4200.48	3000	3000	17,251.03 ab	22,440.48 ab	5676.70 bc	10,774.98 a
I4	F1	1612.62	1758.03	1050.12	3000	3000	13,165.64 c	17,119.09 c	4502.90 cd	8310.93 b
	F2	1612.62	1758.03	2100.24	3000	3000	16,191.98 b	21,368.69 b	6479.12 ab	11,510.42 a
	F3	1612.62	1758.03	3150.36	3000	3000	17,684.14 ab	22,717.24 ab	6921.16 ab	11,808.85 a
	F4	1612.62	1758.03	4200.48	3000	3000	18,284.67 a	24,079.50 a	6471.56 ab	12,120.99 a
I		/	/	/	/	/	***	***	***	***
F		/	/	/	/	/	***	***	***	**
I × F		/	/	/	/	/	***	***	***	***

Note: Iw, irrigation water cost; If, fertilizer input cost; Is, seed input cost; Io, other input cost. I1–I4 represent 60%, 80%, 100%, and 120% *ET_c_*, respectively; F1–F4 represent 70–65–25, 140–130–50, 210–195–75, and 280–260–100 kg ha^−1^ N–P_2_O_5_–K_2_O, respectively. Values are means of three replicates. Different lowercase letters within the same column indicate significant differences at *p* < 0.05. I, F, and I × F indicate the effects of irrigation, fertilization, and their interaction, respectively. ** and *** indicate significance at *p* < 0.01 and *p* < 0.001, respectively.

**Table 7:** Quadratic response surface models of yield, water use efficiency (WUE), and economic benefit as functions of irrigation and fertilization in the 2022–2023 and 2023–2024 growing seasons.

Response	Regression Equation	R^2^	Adj-R^2^	Model
				*p*-Value
2022–2023				
GY	*Y_GY_* = −18,487.433 + 99.131*I* − 2.576*F* − 0.1074*I*^2^ − 0.0104*F*^2^ + 0.0294*IF*	0.98	0.97	<0.001
WUE	*Y_WUE_* = −4.409 + 0.0257*I* − 7.311 × 10^−4^*F* − 2.891 × 10^−5^*I*^2^ − 1.275 × 10^−6^*F*^2^ + 3.759 × 10^−6^*IF*	0.97	0.96	<0.001
EB	*Y_EB_* = −49,260.592 + 228.966*I* − 12.590*F* − 0.2512*I*^2^ − 0.0244*F*^2^ +0.0688*IF*	0.98	0.96	<0.001
2023–2024				
GY	*Y_GY_* = −25,194.658 + 121.796*I* − 3.912*F* − 0.1199*I*^2^ − 0.0119*F*^2^ + 0.0339*IF*	0.98	0.97	<0.001
WUE	*Y_WUE_* = −6.011 + 0.0305*I* − 3.416 × 10^−4^*F* − 3.149 × 10^−5^*I*^2^ − 1.851 × 10^−6^*F*^2^ + 3.872 × 10^−6^*IF*	0.99	0.98	<0.001
EB	*Y_EB_* = −65,459.393 + 284.439*I* − 15.795*F* − 0.2830*I*^2^ − 0.0281*F*^2^ + 0.0800*IF*	0.98	0.98	<0.001

Note: GY, WUE, and EB represent grain yield, water use efficiency, and economic benefit, respectively; I and F represent irrigation amount (mm) and fertilizer rate (kg ha^−1^), respectively. R^2^ is the coefficient of determination, and Adj-R^2^ is the adjusted coefficient of determination. *p* < 0.001 indicates that the regression model is highly significant.

**Table 8:** TOPSIS comprehensive ranking of water–fertilizer treatments in two growing seasons.

	2022–2023		2023–2024	
Rank	Treatment	*C_m_*	Treatment	*C_m_*
1	I3F3	1	I3F3	0.99
2	I3F2	0.936	I3F4	0.888
3	I4F3	0.886	I3F2	0.883
4	I4F4	0.874	I4F4	0.881
5	I4F2	0.851	I4F3	0.873
6	I3F4	0.849	I4F2	0.856
7	I3F1	0.751	I3F1	0.735
8	I4F1	0.724	I4F1	0.708
9	I2F2	0.669	I2F2	0.665
10	I2F1	0.661	I2F1	0.642
11	I2F3	0.534	I2F3	0.563
12	I2F4	0.406	I2F4	0.442
13	I1F1	0.301	I1F1	0.228
14	I1F2	0.225	I1F2	0.179
15	I1F3	0.119	I1F3	0.093
16	I1F4	0	I1F4	0

Note: TOPSIS was performed using treatment means of grain yield, WUE and net economic benefit. *C_m_* represents the relative closeness coefficient; higher *C_m_* values indicate better comprehensive performance.

## Data Availability

The raw data supporting the conclusions of this article will be made available by the authors on request.

## Supplementary Materials

The following supporting information can be downloaded at https://www.mdpi.com/article/10.3390/plants15111629/s1. Table S1: Combined ANOVA for the effects of year, irrigation, fertilization, and their interactions on dry matter accumulation at maturity and crop evapotranspiration of winter wheat across the two growing seasons; Table S2: Combined ANOVA for the effects of year, irrigation, fertilization, and their interactions on leaf net photosynthetic rate (Pn) of winter wheat at different growth stages across the two growing seasons; Table S3: Combined ANOVA for the effects of year, irrigation, fertilization, and their interactions on leaf transpiration rate (Tr) of winter wheat at different growth stages across the two growing seasons; Table S4: Combined ANOVA for the effects of year, irrigation, fertilization, and their interactions on leaf photosynthetic water use efficiency (PWUE) of winter wheat at the anthesis and grain-filling stages across the two growing seasons; Table S5: Combined ANOVA for the effects of year, irrigation, fertilization, and their interactions on output and economic benefits of winter wheat across the two growing seasons.
