# Supplementary material for: Optimized Irrigation and Fertilization Reduce Luxury Transpiration While Improving GRAIN Yield, Water Use Efficiency, and Economic Benefits of Winter Wheat in the Arid Region of Xinjiang
Source: Plants (Basel). 2026 May 26;15(11):1629. doi: 10.3390/plants15111629 (PMC13259080; doi:10.3390/plants15111629)
Supplement: Supplementary file 1 [file plants-15-01629-s001.zip › plants-4331773-supplementary.pdf]

Table S1. Combined ANOVA for the effects of year, irrigation, fertilization, and their interactions on dry matter accumulation at maturity and crop evapotranspiration of winter wheat across the two growing seasons

Source of variation	Dry matter accumulation during maturity （kg ha^{-1}）	Crop evapotranspiration（mm）
Irrigation (I)	***	***
Fertilization (F)	***	***
Year (Y)	***	***
I × F	***	***
I × Y	***	***
F × Y	ns	*
I × F × Y	ns	ns

Note: I, F, and Y represent irrigation, fertilization, and year, respectively. I × F, I × Y, F × Y, and I × F × Y represent their interaction effects. ns indicates no significant difference; *, *** indicate significance at $P < 0.05$, $P < 0.001$, respectively.

Table S2. Combined ANOVA for the effects of year, irrigation, fertilization, and their interactions on leaf net photosynthetic rate (Pn) of winter wheat at different growth stages across the two growing seasons

Pn (μmol m^{-2} s^{-1})				
Source of variation	Green-up	Jointing	Anthesis	Grain filling
Irrigation (I)	**	***	***	***
Fertilization (F)	ns	***	***	***
Year (Y)	***	***	***	ns
I × F	ns	***	***	***
I × Y	ns	ns	ns	ns
F × Y	ns	ns	**	ns
I × F × Y	ns	***	*	**

Note: I, F, and Y represent irrigation, fertilization, and year, respectively. I × F, I × Y, F × Y, and I × F × Y represent their interaction effects. ns indicates no significant difference; *, **, and *** indicate significance at $P < 0.05$, $P < 0.01$, and $P < 0.001$, respectively.

Table S3. Combined ANOVA for the effects of year, irrigation, fertilization, and their interactions on leaf transpiration rate (Tr) of winter wheat at different growth stages across the two growing seasons

Tr (mmol m^{-2} s^{-1})				
Source of variation	Green-up	Jointing	Anthesis	Grain filling
Irrigation (I)	**	***	***	***
Fertilization (F)	*	***	***	***
Year (Y)	**	***	***	*
I × F	*	***	**	***
I × Y	ns	*	ns	ns
F × Y	ns	ns	ns	ns
I × F × Y	ns	ns	***	***

Note: I, F, and Y represent irrigation, fertilization, and year, respectively. I × F, I × Y, F × Y, and I × F × Y represent their interaction effects. ns indicates no significant difference; *, **, and *** indicate significance at $P < 0.05$, $P < 0.01$, and $P < 0.001$, respectively.

`

Table S4. Combined ANOVA for the effects of year, irrigation, fertilization, and their interactions on leaf photosynthetic water use efficiency (PWUE) of winter wheat at the anthesis and grain-filling stages across the two growing seasons

PWUE (μmol CO_2 $mmol^{-1}$ H_2O)		
Source of variation	Anthesis	Grain filling
Irrigation (I)	***	*
Fertilization (F)	***	***
Year (Y)	***	ns
I × F	***	***
I × Y	*	ns
F × Y	ns	ns
I × F × Y	ns	ns

Note: I, F, and Y represent irrigation, fertilization, and year, respectively. I × F, I × Y, F × Y, and I × F × Y represent their interaction effects. ns indicates no significant difference; *, *** indicate significance at $P < 0.05$, $P < 0.001$, respectively.

Table S5. Combined ANOVA for the effects of year, irrigation, fertilization, and their interactions on output and economic benefits of winter wheat across the two growing seasons

Source of variation	Output（CNY ha^{-1}）	Economic benefits（CNY ha^{-1}）
Irrigation (I)	***	***
Fertilization (F)	***	***
Year (Y)	***	***
I × F	***	***
I × Y	***	***
F × Y	ns	ns
I × F × Y	ns	ns

Note: I, F, and Y represent irrigation, fertilization, and year, respectively. I × F, I × Y, F × Y, and I × F × Y represent their interaction effects. ns indicates no significant difference; *** indicate significance at $P < 0.001$, respectively.
